# Association between ALS and retroviruses: evidence from bioinformatics analysis

**DOI:** 10.1186/s12859-019-3249-8

**Published:** 2019-12-20

**Authors:** Jon P. Klein, Zhifu Sun, Nathan P. Staff

**Affiliations:** 1 Department of Neurology, Mayo Clinic, 200 First St. SW, Rochester, MN 55905 USA; 2 Department of Health Science Research, Division of Biomedical Statistics and Informatics, Mayo Clinic, 200 First St. SW, Rochester, MN 55905 USA

**Keywords:** Amyotrophic lateral sclerosis, Retrovirus, Bioinformatics, Pathway analysis, Motor neuron disease, Neurodegeneration, HIV

## Abstract

**Background:**

Emerging evidence suggests retroviruses play a role in the pathophysiology of amyotrophic lateral sclerosis (ALS). Specifically, activation of ancient viral genes embedded in the human genome is theorized to lead to motor neuron degeneration. We explore whether connections exist between ALS and retroviruses through protein interaction networks (PIN) and pathway analysis, and consider the potential roles in drug target discovery. Protein database and pathway/network analytical software including Ingenuity Pathway BioProfiler, STRING, and CytoScape were utilized to identify overlapping protein interaction networks and extract core cluster (s) of retroviruses and ALS.

**Results:**

Topological and statistical analysis of the ALS-PIN and retrovirus-PIN identified a shared, essential protein network and a core cluster with significant connections with both networks. The identified core cluster has three interleukin molecules IL10, Il-6 and IL-1B, a central apoptosis regulator TP53, and several major transcription regulators including MAPK1, ANXA5, SQSTM1, SREBF2, and FADD. Pathway enrichment analysis showed that this core cluster is associated with the glucocorticoid receptor singling and neuroinflammation signaling pathways. For confirmation purposes, we applied the same methodology to the West Nile and Polio virus, which demonstrated trivial connectivity with ALS, supporting the unique connection between ALS and retroviruses.

**Conclusions:**

Bioinformatics analysis provides evidence to support pathological links between ALS and retroviral activation. The neuroinflammation and apoptotic regulation pathways are specifically implicated. The continuation and further analysis of large scale genome studies may prove useful in exploring genes important in retroviral activation and ALS, which may help discover new drug targets.

## Background

Amyotrophic lateral sclerosis (ALS) is a progressive neurodegenerative disorder affecting motor neurons of the brain and spinal cord. To date, approximately 10–15% of ALS cases have been found to have a genetic basis, and causal mutations have been identified in ~70% of familial cases and ~10% of sporadic cases [1, 2]. Causal genes affect diverse mechanisms including protein homeostasis, RNA binding, and cytoskeletal structure [3]. Recently, whole genome and exome sequencing are being applied in large cohorts of ALS patients as pathological causes are discovered at faster pace, not only in coding regions, but also in non-coding regions beyond the currently known C9orf72 repeat expansions [4, 5].

A pathogenic connection between ALS and retroviruses was first suggested by reports of HIV cases presenting either with definitive ALS or ALS-like disease, [6–9] along with the multiple findings that antiviral therapies have been shown to improve some patients ALS-like symptoms [10–12]. In addition, reverse transcriptase, the enzyme utilized by retroviruses, is detected in a significantly higher percent of patients with sporadic ALS over healthy controls [13, 14]. ALS autopsy studies have revealed that human endogenous retrovirus K (HERV-K) expression is significantly elevated in brain tissues of ALS patients [15]. Furthermore, activation of HERV-K genes is noted to kill healthy human neurons grown in cell cultures [16]. Human endogenous retrovirus (HERV) within intergenic regions comprises ~8% of human genome but remains dormant by means of evolutionary selection and is not expressed in healthy persons [17]. Under certain pathological circumstances, endogenous retroviral sequences can become active and expressed [18].

The current hypothesis is that retroviral involvement in ALS could potentially manifest through two routes: exogenous infection by retrovirus akin to human immunodeficiency virus-1 (HIV-1) or endogenous activation of a human endogenous retroviral sequences in the central nervous system such as HERV-K [9]. These two routes may eventually be merged into one overarching mechanism as it is possible exogenous infection could lead to the activation of endogenous retroviral genes embedded in the human genome [19]. More evidence is emerging to suggest a shared pathogenesis between ALS and retroviral activation, and the potential for identifying new drug targets for ALS can be explored through investigating retroviral associated pathways.

Advancement of bioinformatics tools and maturing protein databases now make it feasible to evaluate the shared pathogenesis of ALS and retroviruses using protein network analysis approaches [20]. Because proteins exert their functions through interacting with other proteins, computational topological analysis of the protein network can expedite the identification of essential proteins and may provide the knowledge of biological information that cannot be easily obtained through laboratory experiments, especially for complex diseases like ALS. Herein, we investigated the connections between ALS and retrovirus through protein interaction networks and their associated functional pathways to elucidate the potential pathogenesis connections. Also explored is the possibility of identifying potential drug targets from computerized analyses.

## Methods

### Obtain relevant protein lists using Ingenuity Pathway Analysis Database

We utilized a curated database containing over 6 million findings extracted from scientific publications and public databases, Ingenuity Pathway Analysis (IPA, Qiagen), to obtain two comprehensive lists of proteins implicated in either the ALS or retroviral activation and replication (lists available upon request). These two lists included associated proteins identified through a variety of different scientific methods, including hereditary, genome-wide analysis, animal, and various cell culture functional studies. IPA Annotation, a function within IPA, provides detailed information of the expressed proteins and endogenous biochemical compounds that have been associated with “ALS” or “retrovirus”. We also cross-referenced the Human Genome Mutation Database to ensure that all proteins linked to ALS pathogenesis were included.

### Obtain protein interaction and connection scores

The ALS and retrovirus protein lists generated from IPA were imported into STRING (V.10, string-db.org) [21], an open-source bioinformatics analysis tool for obtaining protein connection scores. STRING uses a score combiner algorithm to determine probability of protein connectedness or relation. The generated combination scores were further rescaled into the confidence range from 0.0 to 1.0, connecting all the scores. Default String confidence scores <0.400 indicate low confidence, 0.400–0.700 indicate medium confidence, and > 0.700 indicates high confidence. STRING allows users to filter out protein interactions based on these scores. To create a relatively stringent yet comprehensive set of interactions, we applied medium confidence protein-protein interactions (STRING scores ≥0.400) as a cut-off filter. The interaction scores and relationships of two sets of proteins (ALS set and Retrovirus set) were then extracted as a text-delimited file (lists available upon request).

### Protein sub-interaction or overlapping network identification and annotation

The protein interaction files from String were imported into the open-source software Cytoscape 3.6 [22] for protein interaction network (PIN) construction, visualization, and network analysis. Cytoscape 3.6 allows users to visualize protein connections and perform topological and statistical analysis, also identify significantly overlapping core protein clusters, or sub-networks, of the two larger PINs. Cytoscape CentiScape, a Cystoscope plug-in function, evaluates the topological properties of the PINs by measuring a series of network parameters, including degree centrality, closeness, and betweenness. In protein networks, the term “degree” indicates the number of edges that a node has, thus the higher the degree, more connections this node has. Betweenness measures how close a node is to all other nodes in the protein network. The topological parameter indicates the relevance of a node (protein) as a functionally connecting link in a biological network. Nodes with a high degree (hub genes) represent proteins with higher relevance in connecting regulatory molecules, suggesting important biological function. For example, networks whose surrounding topologies resembling a star have centralization close to 1, whereas completely decentralized networks are characterized by having centralization close to 0. Node centrality in both networks was investigated by evaluating “node degree”.

Multiple Cytoscape plug-in functions analyze biological functional enrichment to assess degree importance of each protein (node) within the PIN. One of the Cytoscape applications, Molecular Complex Detection (MCODE), was applied to detect densely connected regions that may represent molecular complexes, i.e. important protein subnetworks or clusters. MCODE extracts the dense regions around a protein of interest and provides a level of functional annotation above simple-associations using multiple connectivity algorithms. The MCODE algorithm operates in three stages, vertex weighting, complex prediction and post processing to filter or add proteins in the resulting complexes by means of certain connectivity criteria [23]. Since subunits of a molecular complex generally serve function in the same biological process, relevant clusters can be annotated to understand what molecular pathway are central to the protein network. Another Cytoscape application, ClueGo [24] along with Ingenuity pathway analysis were used to perform biological pathway enrichment analysis for the core cluster identified by MCODE. ClueGO integrates Gene Ontology (GO) terms as well as KEGG/BioCarta pathways and creates a functionally organized pathway term network. ClueGo is one of the most commonly used applications in Cytoscape for biological interpretation and visualizing functionally grouped proteins.

## Results

**Fig. 1:**
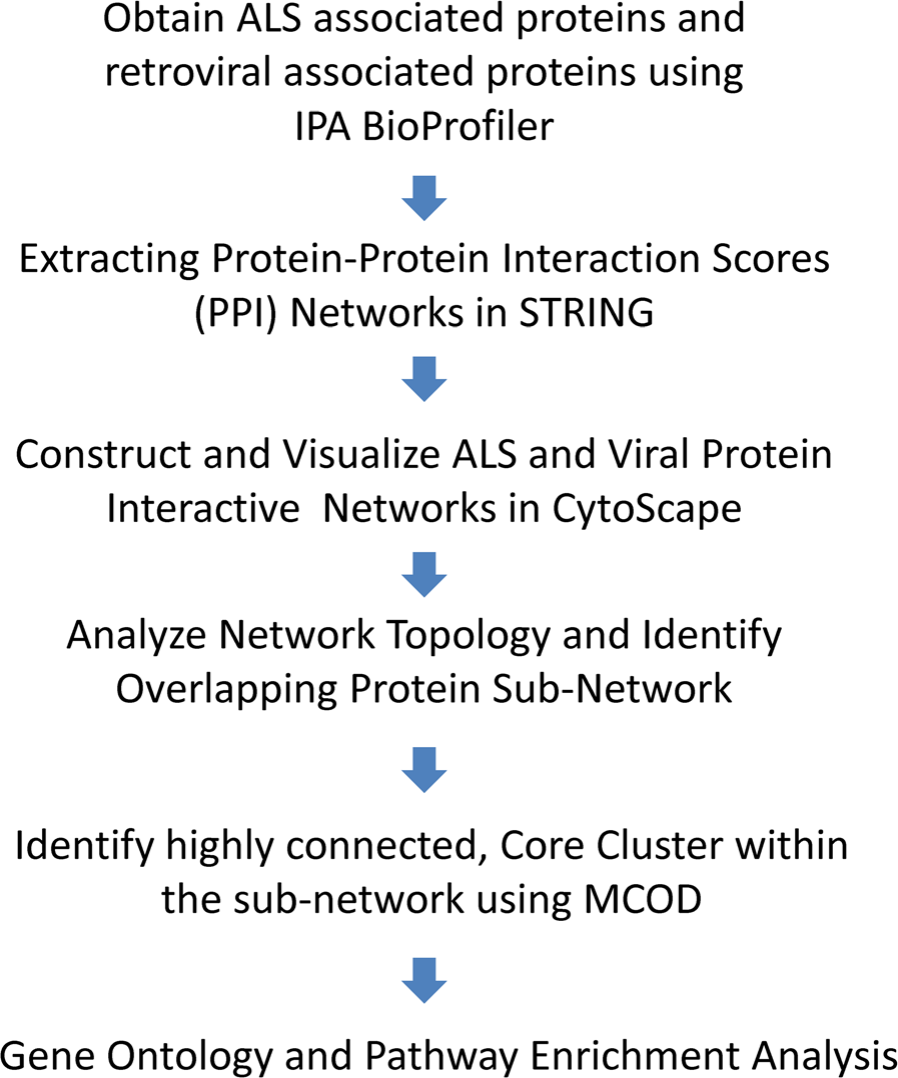
Bioinformatics Study Design

The overall workflow of our study is shown in Fig. 1. The computerized process involves the following steps: 1) Retrieve associated proteins from IPA; 2) Obtain protein connection scores in STRING; 3) Import protein connection score files and construct protein interaction networks in CytoScape 3.6; 4) Conduct network topological and statistical analysis using multiple CytoScape 3.6 Plug-in tools; and 5) Identify existing overlapping protein core cluster (s) that are shared by both networks, 6) perform functional pathway analysis.

### Protein interaction networks of ALS and retrovirus have more than expected connections

**Fig. 2:**
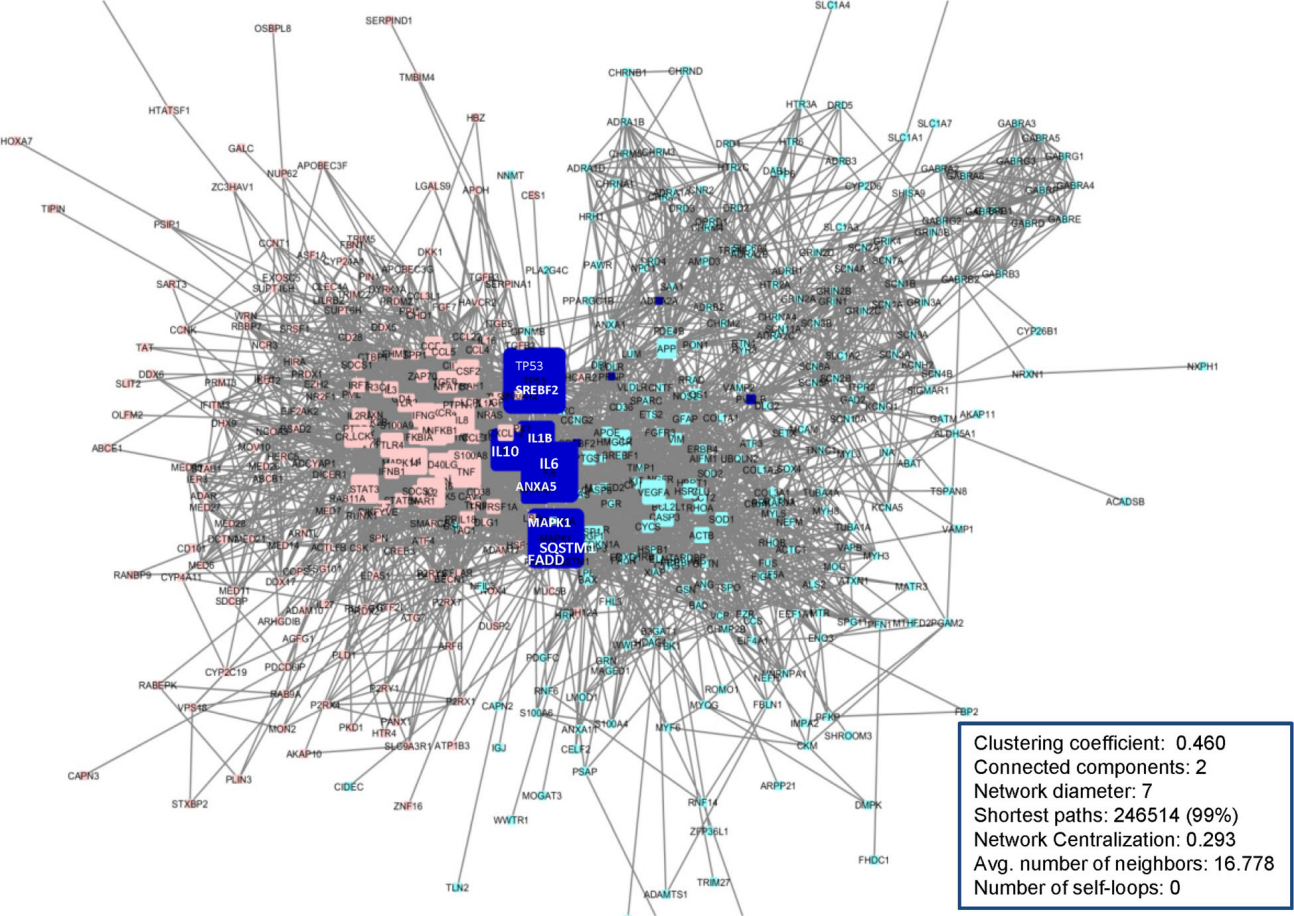
Merge of ALS protein network (cyan colored nodes) and retrovirus protein network (rose colored nodes) with overlap nodes in both networks (blue colored nodes). The size of nodes represents the degree and between-ness of nodes. The protein network topology and statistical analysis by Cytoscape network analyzers showed the importance of the nodes in each network based on a series of topological parameters, including the node centrality measure and node degree. The positions of nodes within this joined protein network are determined by topological parameters derived from Cytoscape’s algorithm for merging networks. The joined network is visualized based on the node degree (represented by node size) and edge betweenness parameters (represented by location and sizes of edges). Values of edge betweenness were mapped with the edge size: high values of this parameter correspond to a large edge size

We constructed two extended PINs in Cytoscape 3.6 to explore the connection between ALS and retroviral activation and the potential association to ALS etiology (Fig. 2). All interactions in the networks are unweighted and undirected, and duplicated edges and self-loops were removed. In both networks, each node represents a protein while edges represent the interactions between proteins. The ALS PIN includes 273 nodes and 1878 edges while the retroviral protein interaction network includes 242 nodes and 2385 edges. The two networks were merged in CytoScape to form a joined network, with cyan color representing nodes within ALS PIN and pink color representing nodes within retrovirus PIN. If a node exists in both networks, its color will become blue. The nodes that are highly connected to both networks will appear larger. The analysis results demonstrated that these two networks have statistical significance of protein association, i.e. significantly more interactions comparing to same number of randomly selected protein interactions (PPI enrichment *p*-value:8.83e-16).

### A shared sub-network is identified between the ALS and retrovirus PINs

**Fig. 3:**
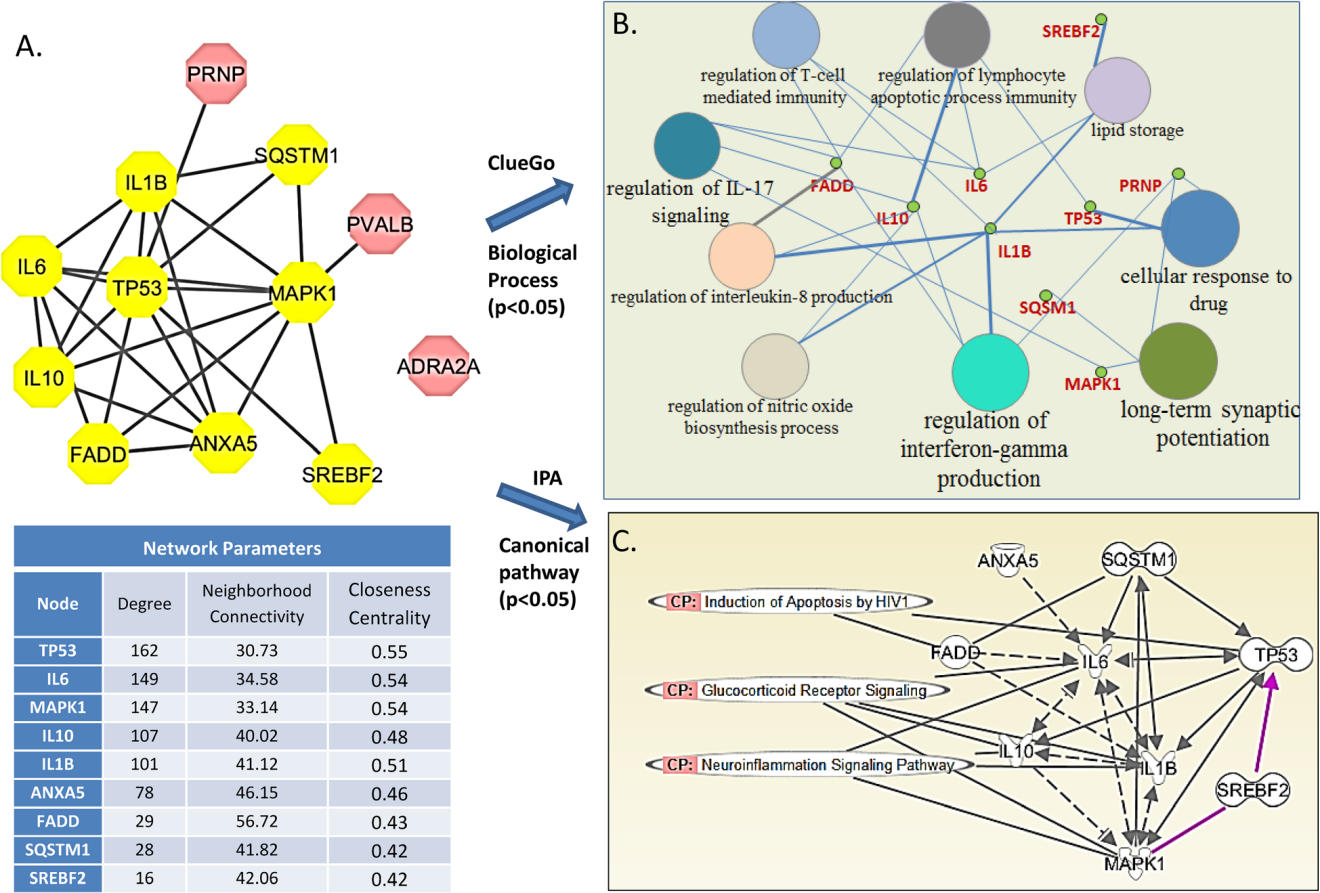
**a** A MCODE Cluster within shared subnetwork of ALS protein network and retrovirus protein network. The nodes (proteins) that are yellow are retained in the MCODE Cluster after analysis, based on their parameters, including Connectivity, Degree and Centrality. The table outlines the degree, neighborhood connectivity and closeness centrality values of the MCODE Cluster Nodes. The neighborhood connectivity of a node is defined as the average connectivity of all neighbors. The closeness centrality is a number between 0 and 1 and measures how fast information spreads from a given node to other nodes in the network. The rose colored nodes are within shared subnetwork but excluded from the MCOD Cluster. **b**. Clue-Go analysis identifies KEGG pathways and biological processes linked to the essential proteins in the Core Cluster. **c**. IPA pathway analysis identifies canonical pathways linked to the Core Cluster proteins

Next, we applied Cytoscape’s network analysis algorithm “intersect” to extract subnetworks that are shared in both ALS and retroviral protein networks, in other words, this is to identify the nodes that are not only highly connected (indication of functional importance and relevance), but also showing similar relevance and importance for both networks. The high-degree nodes (proteins) presented in the shared sub-networks of ALS and retrovirus could provide directions for exploring drug targets. The intersected (overlapping) networks were obtained and extracted as an independent new network in Cytoscape. A distinct overlapping protein interaction network of ALS and retroviral expression/activation network (PPI enrichment *p*-value:1.02e-4), consisting of 12 nodes and 26 edges, emerged (Fig. 3). The fact that we were able to identify an overlapping sub-network that showed highly connected edges to both networks suggests that common pathways involved in the pathogenesis of both disorders likely exist.

### MCOD Identify a core cluster within shared sub-network

In order to closely examine densely interconnected core cluster within the overlapping sub-network that may represent complexes playing essential role in both PINs, we applied Cytoscape MCOD (Molecular Complex Detection) algorithm to identify the densely connected MCOD Clusters (Fig. 3) [23]. MCOD Clusters represent closely connected protein complexes that often share same functional pathways. The MCOD algorithm utilizes a directed mode that allows fine-tuning of clusters of interest and allows examination of cluster interconnectivity relevant for protein networks. We identified a MCOD Cluster within the overlapping ALS and retroviral protein networks with MCOD score of 6.333. The MCOD Score is defined as the product of complex subgraph density and number of nodes (proteins) in the complex subgraph, and a score of > = 6 indicates 90% predictive accuracy [23]. Proteins (nodes) of this core cluster include three major interleukins IL10, Il-6 and IL-1B and central apoptosis regulators TP53, MAPK1, ANXA5, SQSTM1, SREBF2, FADD (Fig. 3a). The highly connected proteins within the core cluster appeared to be essential elements in overlapping protein network of viral activation and ALS. The overlapping sub-network also included PRNP, PVALB and ADRA2A, but none of them had enough edges connected to other nodes to qualify them in the core cluster.

### Biological pathway and gene ontology analysis

Ingenuity Pathway Analysis (Qiagen) and the Cytoscape plug-in Clue-Go were utilized to identify associated biological functional pathways. The Clue-Go enrichment analysis identified significantly enriched biological function processes and IPA identified canonical signaling cascades that are associated with the identified nodes from the core cluster. ClueGo showed this core cluster networks is significantly associated with a number of Biological Process, including regulation of interleukin production, neuron apoptosis process, and interferon gamma production (Fig. 3b). IPA linked three relevant canonical pathways to the core cluster, induction of apoptosis by HIV, glucocorticoid receptor signaling, and neuroinflammation signaling pathway (Fig. 3c).

### Associations between ALS, HIV, Polio and West Nile virus protein interaction networks

**Fig. 4:**
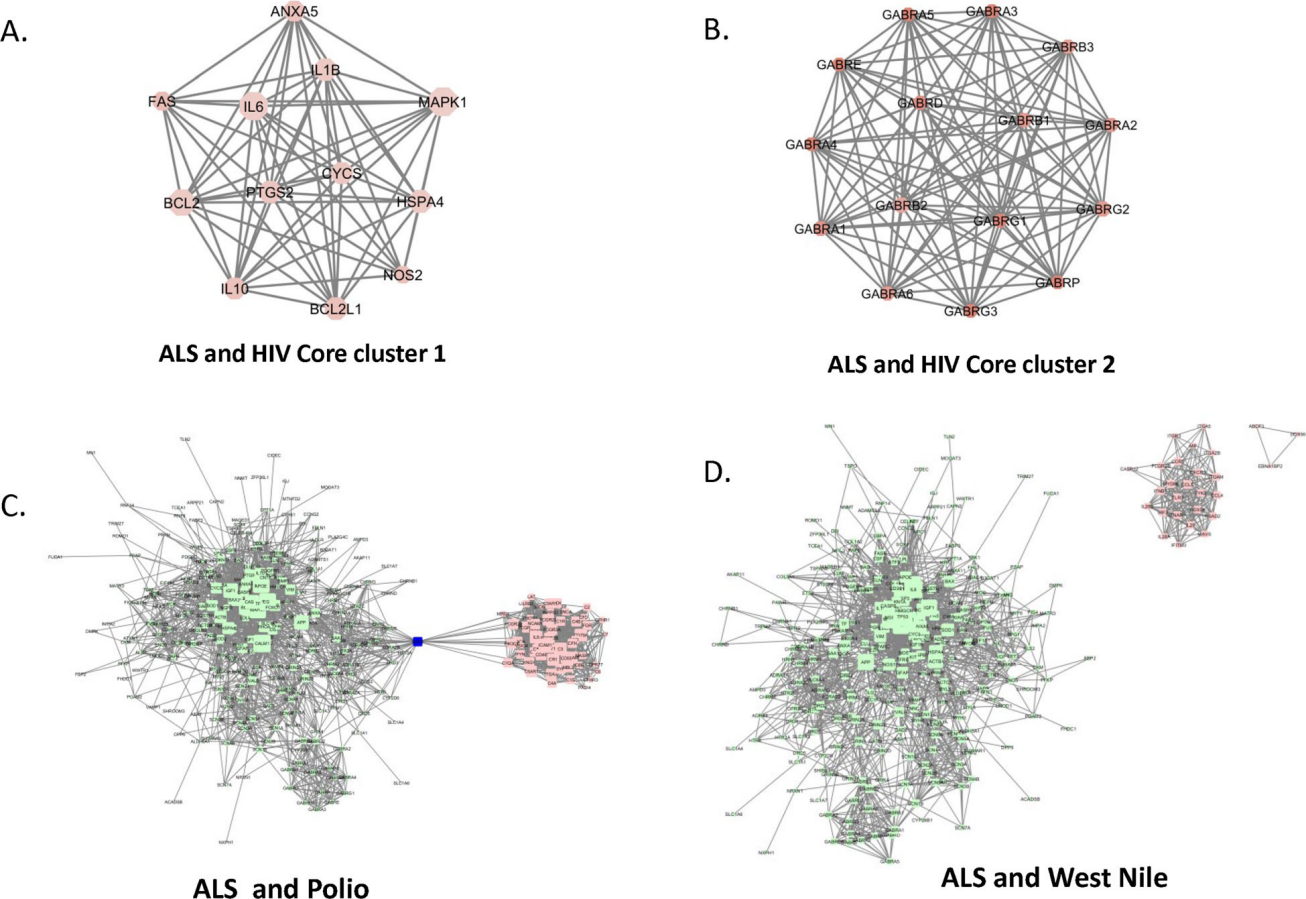
**a-b** Two core clusters are identified from ALS-HIV merged PINs. Cluster-1 also contains three interleukins, IL6, Il10 and Il-1B. Cluster-2 composed of all GABA signaling receptors. **c**. ALS-Poliovirus merged PINs, there is only one shared protein, DRD2, but there is no overlapping network. **d**. ALS-West Nile Virus merged PINs, there is no overlapping protein nor network

As evidence has shown associations between HIV and ALS, we applied the same analytical approach to generate a PIN for HIV. The HIV protein network contains 1084 of nodes and 15,715 edges, after merging with ALS PIN, two highly connected, statistically significant overlapping cluster emerged from the combined PIN. One of them shared similar nodes found from the ALS-retrovirus cluster such as IL6, IL1B, Il10, MAPK1, ANXA5; the second overlapping core cluster is composed of all GABA receptors, forming a highly connected sub-network themselves (Fig. 4a-b). Because of the potential connections of motor neuron disease with Poliovirus and West Nile virus, we also investigated whether there is any significant overlapping network (s) existing between Poliovirus, West Nile virus and ALS networks, but no overlapping networks were found between these networks (Fig. 4c-d). These results underscore the unique connections between ALS and retrovirus/HIV protein networks.

## Conclusions

In this study, we provide bioinformatics evidence to support a disease pathogenesis connection of ALS and retroviral activation/replication. We performed topological and statistical analysis of protein networks and uncovered a core protein cluster shared between protein networks of ALS and retrovirus. We explored the canonical pathways and biological processes that are associated with the proteins in the core cluster and investigated whether the shared protein core cluster of ALS and retrovirus networks could suggest useful directions for exploring rational drug targets. ALS is a devastating neurodegenerative disease, but currently there is no effective treatment. Currently, there are a number of treatments available for retroviral related disorders such as HIV, searching potential drug targets may help identify existing anti-viral drugs that can potentially be repurposed to treat ALS patients, or direct future drug developments.

The presence of a shared protein core cluster strongly suggests the existence of overlapping molecular pathways. The identified cluster contained three major cytokines from the interleukin family (IL1B, IL6, and IL10), and six critical regulators of cytokines and cell apoptosis signaling (SQSTM1, TP53, MAPK1, ANXA5, SREBF2, and FADD). SQSTM1 is one of the ALS causal genes and an autophagic receptor that recognizes and transfers ubiquitinated proteins for autophagic degradation. It is interesting that cytokines of the interleukin family emerged from protein networks with the highest node-degrees and prominent connections to both ALS and retroviral PINs. Interleukins are key players of pro-inflammatory and anti-inflammatory homeostasis. They are vital for retrovirus-related disease mechanisms and neuroinflammation. Interestingly, immune response has been suggested to play an important role in both neuroprotection and neurotoxicity [25, 26].

ALS pathogenesis involves impairments of divergent cellular functions, including protein aggregation, RNA processing, protein ubiquitination, glutamate toxicity, mitochondrial dysfunction, and axonal transport [3]. However, the exact mechanism of how these divergent biological processes eventually converge into motor neuron degeneration has not been defined. Our protein network analysis supports that neuroinflammation may be the converging disease mechanisms for ALS, or plays critical roles impacting the progression rate of ALS, if not the cause of the disease [27]. Previous studies have shown increased levels of circulating cytokines in ALS cases. ALS pathology studies have also shown morphological evidence of microglial activation [28, 29] .

Proinflammatory cytokine IL-6’s immunoreactivity has been found significantly upregulated in frontal cortex of ALS cases [30]. Another proinflammatory cytokines IL-1*β* produces structural damage to neurons and causes neuronal dysfunction by acting on glutamate-mediated excitatory postsynaptic currents [31, 32]. Increased numbers of astrocytes and activated microglia have also been observed in the anterior horn of the spinal cord and pyramidal tracts of ALS cases. On the other hand, IL-10, an anti-inflammatory cytokine, decreases secretion of pro-inflammatory cytokines such as IL-6, and controls differentiation and proliferation of macrophages, T cells, and B cells. Thus, IL-10 may protect against excessive immune responses and tissue damage, keeping pro-inflammatory events under control [33]. The timing and anatomical localization of immune responses will be important in understanding how the immune response functions in disease pathogenesis, as cytokine signaling likely triggers different pathways depending on different disease stages. Interestingly, a recent study also linked Superoxide Dismutase 1 (SOD1), the first identified ALS causal gene, to inflammatory mechanism. The expression of mutant SOD1 in astrocytes and microglia contributes to disease progression in ALS through IL10, as IL-10 controls early microglial phenotypes and disease onset in ALS caused by misfolded SOD1 [34].

Almost all ALS patients (>90%) have an accumulation of protein and protein-RNA aggregates in the autopsy spinal cord and brain no matter the specific cause of the disease [35]. These aggregates are toxic to cells and usually formed by proteins encoded by TAR-DNA binding protein TARDBP *(TDP-43)*. Although TARDBP is not one of proteins in the shared core cluster, it is critical in processing IL-6 and IL-10 at the nuclear site for cytokine RNA production within IL6 and Il10’s splicing activating compartment, suggesting TDP43 pathogenesis could converge into neuroinflammation pathway [36].

In silico protein network analysis has been gaining attention in drug discovery through identifying the potential drug targets. The drug discovery process usually starts with discovering essential proteins (drug targets). The proteins in the ALS-retrovirus PIN core cluster can be putative drug targets for experimental validation by cell culture, animal testing and eventually clinical trials in patients. Interestingly, IL-6 has already been selected as the target and a phase 2 randomized, placebo controlled trial of Tocilizumab (targeting IL-6) in ALS patients is currently in data analysis stage. It is rational to consider this approach can be applied in other diseases.

## Data Availability

The datasets used and/or analyzed during the current study are available from the corresponding author on reasonable request.

## Abbreviations

ALS: Amyotrophic lateral sclerosis
HERV: Human endogenous retrovirus
HIV-1: Human immunodeficiency virus-1
MCODE: Molecular complex detection
PIN: Protein interaction network
SOD1: Cu/Zn superoxide dismutase 1
